# Evaluating a novel core-and-perimeter delimiting trapping survey design for insects. I. Field experiment

**DOI:** 10.1093/jee/toaf095

**Published:** 2025-06-14

**Authors:** Barney P Caton, Hui Fang, Ernie Hain, Nadya Kandel, Rosalie Nelson, Godshen R Pallipparambil, Nicholas C Manoukis

**Affiliations:** Plant Protection and Quarantine, Animal and Plant Health Inspection Service, United States Department of Agriculture, Raleigh, NC, USA; Center for Integrated Pest Management, North Carolina State University, Raleigh, NC, USA; Plant Protection and Quarantine, Animal and Plant Health Inspection Service, United States Department of Agriculture, Raleigh, NC, USA; Center for Integrated Pest Management, North Carolina State University, Raleigh, NC, USA; USDA Agricultural Research Service, Daniel K. Inouye U.S. Pacific Basin Agricultural Research Center, Hilo, HI, USA; USDA Agricultural Research Service, Daniel K. Inouye U.S. Pacific Basin Agricultural Research Center, Hilo, HI, USA; Center for Integrated Pest Management, North Carolina State University, Raleigh, NC, USA; USDA Agricultural Research Service, Daniel K. Inouye U.S. Pacific Basin Agricultural Research Center, Hilo, HI, USA; USDA Agricultural Research Service, European Biological Control Laboratory (EBCL), 810 avenue du Campus Agropolis 34980 Montferrier-sur-Lez, France

**Keywords:** boundary, dispersal, quarantine, surveillance, invasive pest

## Abstract

We propose a novel “core-and-perimeter” delimiting trapping design for invasive insects, improving upon the ubiquitous fully trapped square grids using regular spacing. The core-and-perimeter design has traps near the epicenter and in a perimeter set at a distance to result in zero captures, to directly set the population boundary. We compared the core-and-perimeter and fully trapped designs in a mark–release–recapture experiment with *Ceratitis capitata* (Weidemann) (Diptera: Tephritidae) in Hawaii in 2022. Each design had 4 repetitions with 4 separate releases of flies and 6 collection days from 1 to 14 d after release. The square fully trapped grid had 20 core area traps plus 79 other traps in 0.92 km^2^, plus 24 “sentinel” traps beyond. The circular core-and-perimeter grid had 20 core traps, and 108 traps in a 220 m-wide perimeter, set 500 m from the release point (smaller than the recommended radius). Slightly more flies on average were captured in the fully trapped treatment but proportional captures in common locations were similar. Flies were caught in the sentinel traps in every replicate of the fully trapped treatment. Four percent of captures occurred in the perimeter of the core-and-perimeter treatment on average. Trap usage rate for the fully trapped design was 67%, while for the core area of the core-and-perimeter design was nearly 94%. Overall mean daily dispersal distance was 96.3 m, and the regression-based 99th percentile of total distance was 700 m. Results supported the potential of the core-and-perimeter design and demonstrated 2 fully trapped design disadvantages—trap inefficiency and egress potential.

## Introduction

Delimiting surveys are often an important early step in the response to the discovery of new nonendemic plant pests in the United States and elsewhere (Jang et al. 2014, van Havre and Whittle 2015). They are conducted to establish the boundaries of the infested area (IAEA 2003, IPPC 2018), with the specific purposes of confirming the presence of the pest population and determining the extent of the area occupied by the population (van Havre and Whittle, 2015). Delimitation is critical for decision-making and management success (Mangel et al. 1984, Panetta and Lawes 2005), and the methodology should “allow for the *confident* identification of the boundaries of an [infested] area” (Plant Health Australia 2015). The second objective largely determines how extensively the eradication or management response may need to be applied (Tobin et al. 2014), affecting eradication costs as these are largely a function of the total area managed (Woldendorp and Bomford 2004, Harris and Timmins 2009). Hence, accuracy is important for avoiding underestimation (lack of containment), or overestimation (to constrain costs).

**Fig. 1.**
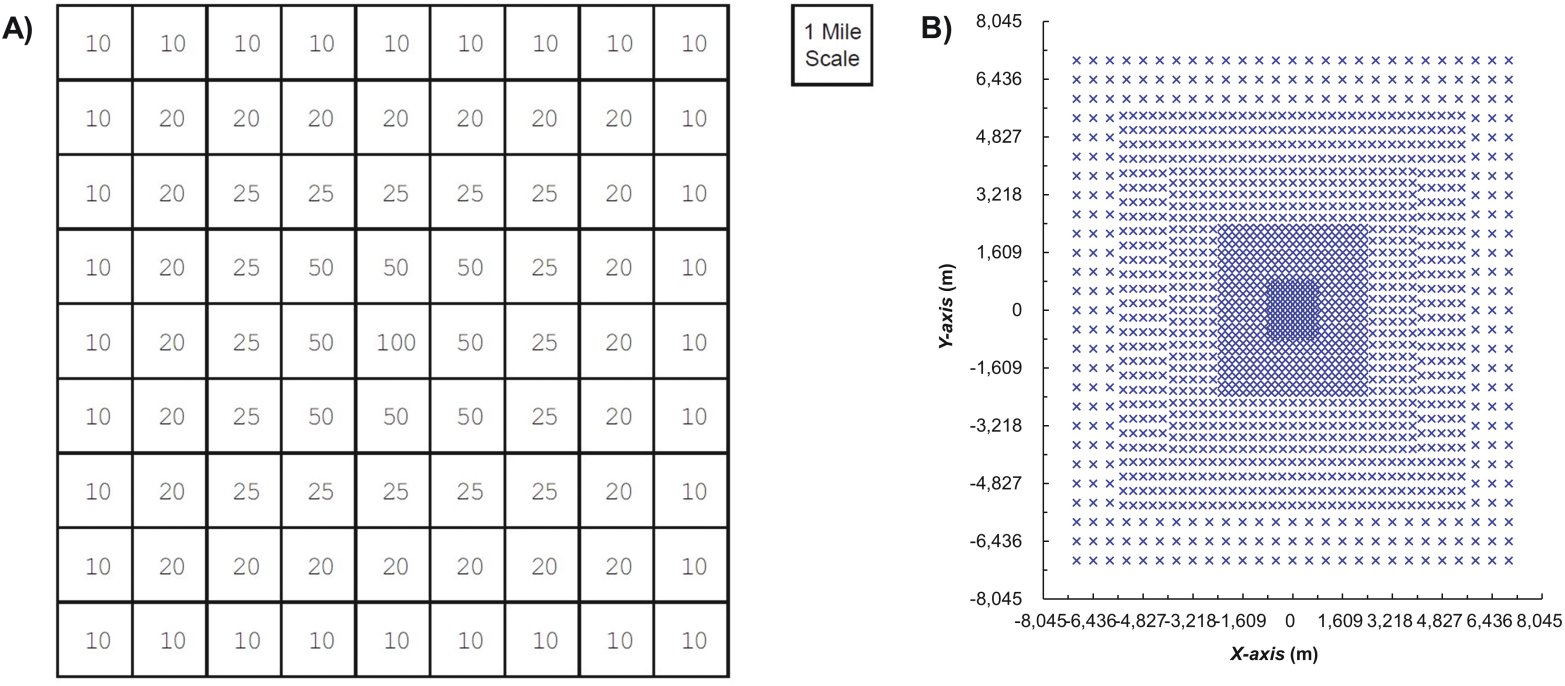
Published delimitation trapping survey grids for Medfly, showing A) numbers of traps per cell in the basic design with a 14.5-km side length (PPQ 2003, CDFA 2013), and B) a grid version with 1,660 trap locations.

Many insect delimiting activities involve trapping (CDFA 2013, Meats 2014, Weldon et al. 2014). For example, the published plan for the Mediterranean fruit fly (Medfly, *Ceratitis capitata* (Weidemann) [Tephritidae: Diptera]) is a 14.5 km (9 mi) square with 1,700 traps (Fig. 1; PPQ 2003, CDFA 2013). Every delimiting trapping survey design we have found for use in the United States has employed square grids with traps spaced regularly throughout at a specified density (or densities as in the example of Medfly, above). We refer to these square trapping grids as “fully trapped” (FT) designs. “Survey design” in the case of delimitation of a new pest in the United States typically consists of choosing a grid size—often either 8 km (5 mi) or 14.5 km (9 mi)—and then specifying trap densities throughout (uniform or variable).

Delimitation trapping designs have often relied on the experience and intuition of local managers (Roberts and Ziegler 2007, Jang et al. 2014), which was apparently true for the expert group that created the Medfly design in the 1980s (PPQ, personal communication). Our understanding is that the group addressed grid size uncertainty by multiplying the estimated side length by a factor of 3 and probably incorporated variable densities (Fig. 1) to reduce the number of traps used. These designs, and the principles behind them, had not been rigorously studied until recently. Many FT designs were likely created using limited scientific information, yet have been used for decades without changes. By contrast, trapping networks for pest *detection*, also known as “detection surveys” (IPPC 2024), have been studied fairly extensively (Epanchin-Niell et al. 2014, Meats 2014, Berec et al. 2015, Yemshanov et al. 2017).

Recent empirical (Dominiak and Fanson 2020, Caton et al. 2021a) and simulation studies (Caton et al. 2021b, Dominiak and Fanson 2023) suggest that many delimitation trapping areas are oversized and that trap densities could be better optimized by accounting for trap and lure attractiveness. For example, compared to the standard FT design for Medfly, a 4.8 km diameter circular grid with only 232 traps still yielded a reasonable *p*(Detection) and reduced the cost by 86% (Fang et al. 2022). Another study indicated that quarantine areas for *Bactrocera tryoni* (Froggatt) (Diptera: Tephritidae) (Queensland fruit fly) were similarly oversized (Dominiak and Fanson 2023). Trapping grids have likely been oversized because of limited information on pest dispersal abilities. Recent dispersal analyses, discussed below, have confirmed that delimitation or quarantine trapping radii for several fruit fly species could be shortened. Thus, current approach that has dominated delimitation trap network design may often generate oversized grids that may not provide the expected level of *p*(Detection).

Properly sizing delimitation grids would improve cost efficiency, but the FT design has at least 2 inherent disadvantages. The first is that many more traps are used and serviced than the number of insects captured. In a recent dispersal distance analysis (Caton et al. 2021a), the mean total number of captures per population cluster was 4.6 for *Bactrocera dorsalis* (Hendel) (Oriental fruit fly; Diptera: Tephritidae) in California and Florida, and 2.7 for *Anastrepha ludens* (Loew) (Mexican fruit fly; Diptera: Tephritidae) in Texas. The same metric was 7.7 for Medfly in Florida, which equals a rate of 0.0045 flies per trap for the standard FT grid. Even in mark–release–recapture studies, recapture rates are often low. In 4 studies on the Queensland fruit fly with release rates from 320,000 to 45 million flies, the mean recapture rate was only 0.10% (Dominiak 2012). The total number of traps and their distribution is important because trap servicing, or periodic checks for captured insects and replacing or replenishing lures (eg IAEA 2003), can incur considerable costs (Florec et al. 2013). Trapping programs are often constrained by staffing resources or budgets (Marsh and Trenham 2008, Quilici and Donner 2012, Lance 2014, Schellhorn and Jones 2021), so limiting the number of traps—and associated servicing travel and costs—could be important.

**Fig. 2.**
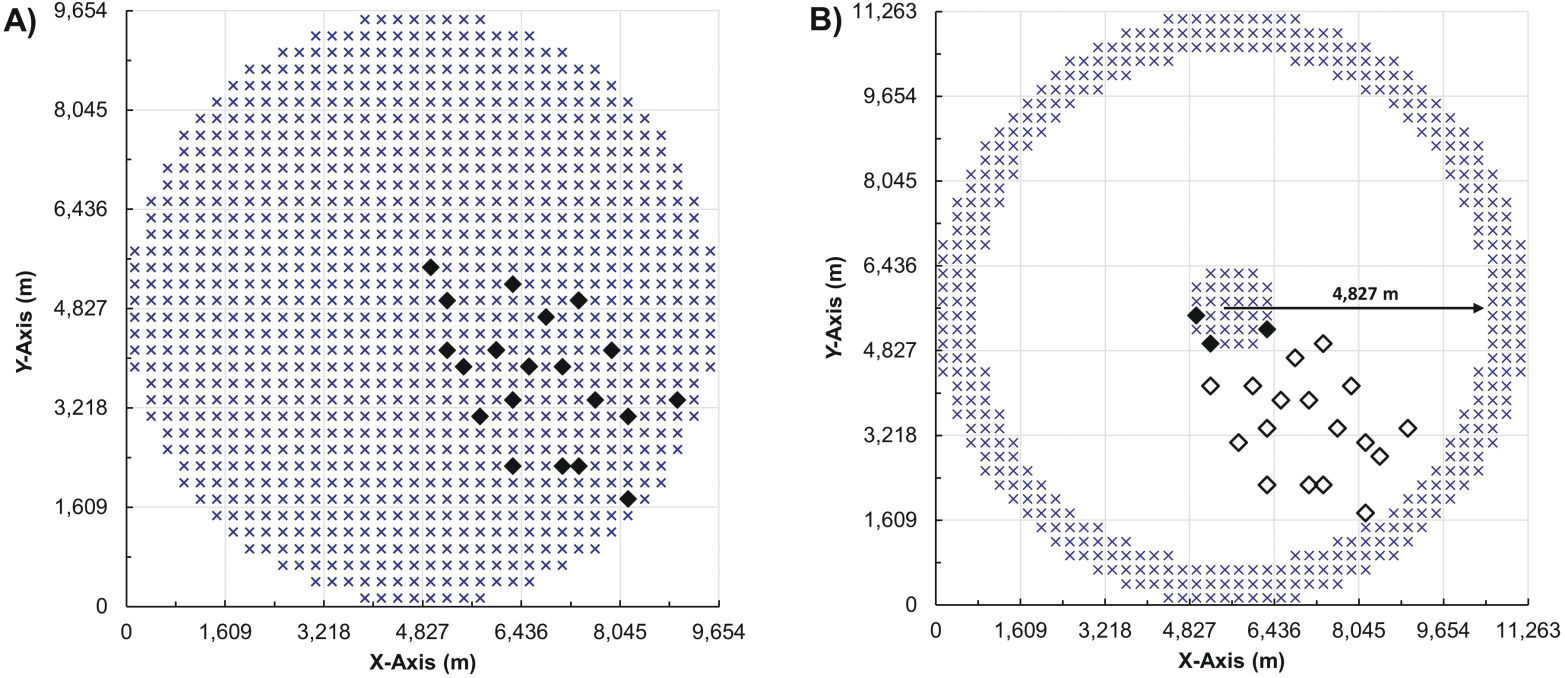
Twenty hypothetical insect locations in A) a FT delimiting survey grid with a 4,800 m (3 mi) radius and 1,020 traps (blue Xs), and B) a C&P delimiting survey grid with a 4,827 m (3 mi) inner perimeter radius and 408 total traps (Xs). Shown are hypothetical captures (solid diamonds) and noncaptures (empty diamonds). The trap density in both designs is 13.9 traps per km^2^.

The second disadvantage of FT designs is that insects can depart the grid without notice. Captures near grid boundaries provide good evidence that pests *may have* escaped the area (Fig. 2A). In a real-world example, third-generation *Uraba lugens* (Walker) (Lepidoptera: Nolidae) individuals were detected in several traps along the southern and western boundaries of the delimitation area in New Zealand (Suckling et al. 2005). The standard remedy for this flaw is to increase the grid size beyond the expected dispersal distance to reduce the likelihood of undetected egress. This size inflation increases costs significantly. All else being equal the C&P designs also may experience escapes from the grid, but if sized appropriately a lack of captures in the perimeter can provide helpful direct information on spread extent (Fig. 2B).

**Fig. 3.**
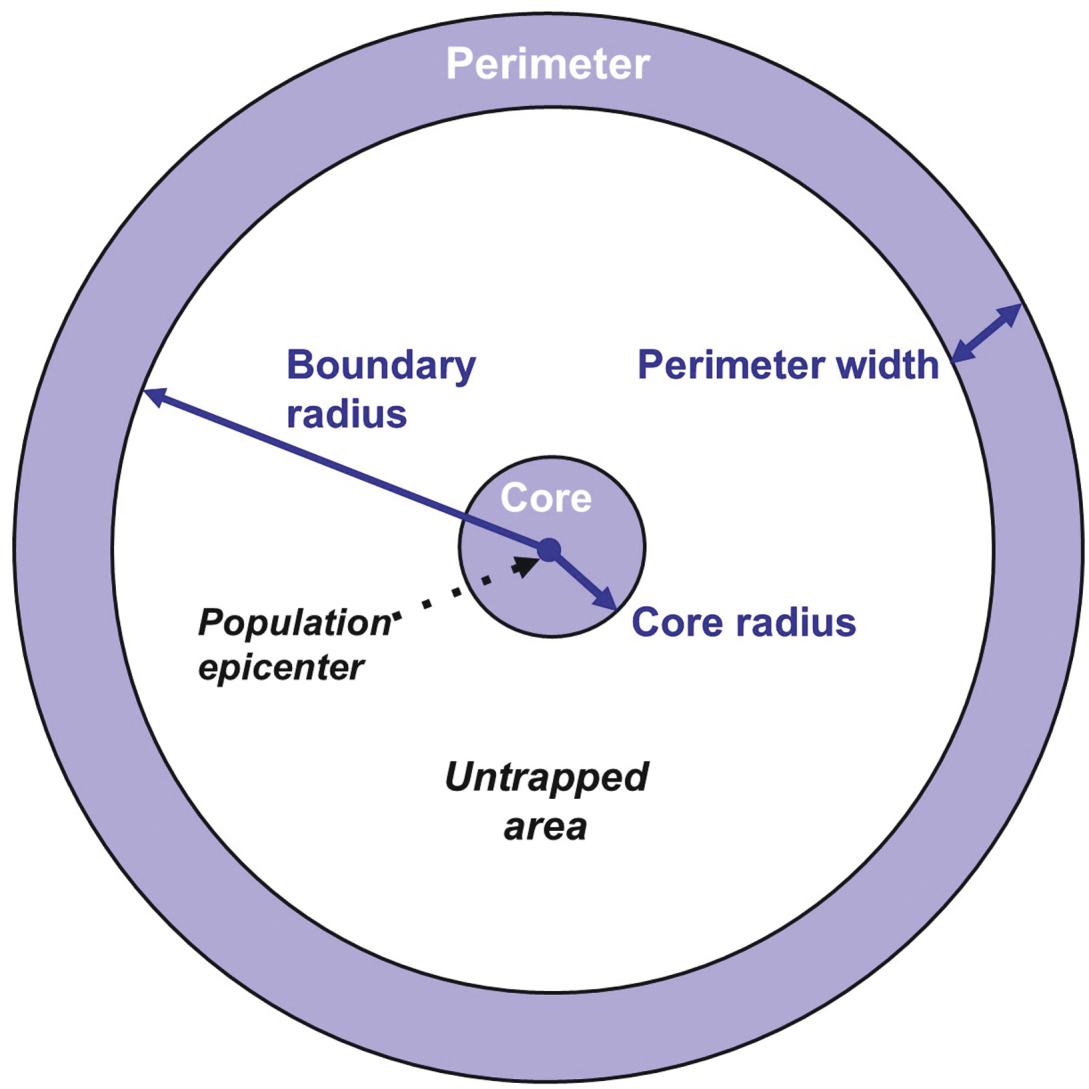
A C&P delimiting survey design, set around the epicenter of an insect population. Traps are found only in the core and perimeter areas (shaded areas). The core has a defined radius of 800 m, and the perimeter is set a certain distance outward depending on species dispersal ability, with a fixed width of 800 m.

To improve delimitation trapping we propose a “core-and-perimeter” (C&P) plan, in which traps are placed within a central, core area, and an outer perimeter (Figs. 2B and 3). The core area is centered on the initial detections (presumed epicenter), and the traps there are meant to confirm the presence of the pest population (Goal 1 above). Captures are statistically most likely to occur near the source, where population density is greatest (Meats 1998). The perimeter traps are placed away from the epicenter at an optimized distance that is unlikely to result in captures. A pest-free perimeter avoids problematic captures near the boundary (Fig. 2B) and provides evidence of containment and accurate delimitation (Goal 2). Traps placed between the 2 areas might provide additional pest captures but those are unlikely to contribute much to the delimitation goals. Eliminating those traps makes the C&P design more efficient for delimitation grids with inner perimeter radii of 3.2 km (2 mi) or more (Table 1). The benefits of the C&P design are improved containment, more certain trapping outcomes, and substantial savings in trap and servicing costs, especially for larger grids.

**Table 1.** Grid areas by boundary radius for FT and C&P designs, showing numbers of total traps, and proportional trap savings for the C&P approach. The density used was 13.9 traps per km^2^ (36 per mi^2^)

Boundary radius (km) [mi]	Fully trapped		Core-and-perimeter			Proportional trap reductions
	Area (km^2^)	Traps (no.)	Perimeter area (km^2^)	Total area (km^2^)	Traps (no.)	
1.6 [1]	8.1	113	5.8	7.9	190	-0.68
2.4 [1.5]	18.3	254	8.4	10.4	261	-0.03
3.2 [2]	32.5	452	10.9	12.9	331	0.27
4.0 [2.5]	50.8	707	13.4	15.4	401	0.43
4.8 [3]	73.2	1,017	15.9	18.0	471	0.54
5.6 [3.5]	99.6	1,385	18.5	20.5	542	0.61
6.4 [4]	130.1	1,809	21.0	23.0	612	0.66
7.2 [4.5]	164.7	2,289	23.5	25.5	682	0.70
8.0 [5]	203.3	2,826	26.1	28.1	752	0.73

Because the C&P design is so different from the FT plans currently in use, field evaluation is important. We conducted mark–release–recapture experiments with Medfly near Hilo, Hawaii in 2022 to compare captures in the FT and C&P designs and to evaluate if the C&P design performed as expected and is likely to improve delimitation outcomes. Because of the limited area available for the experiment, the C&P design tested was smaller than the recommended size for Medfly. Therefore, the field trials were not a direct test of the worthiness of the C&P design but did provide information on operations and Medfly dispersal distances. We report here on the trapping outcomes for the 2 designs, spatial comparisons, and Medfly dispersal, emphasizing design performance, and checking for potential design flaws. We used that information to calibrate a correlated random-walk dispersal model for Medfly, which we validated with independent data, and then used separately (Caton et al. 2024) to more continue testing the C&P design.

## Methods

### Field Site

Experiments were conducted in the Island Princess (IP) macadamia orchard in Keaau, East Hawaii Island (hereafter IP; 19 36.725N, 155 05.084W). The IP site has tall macadamia trees (*Macadamia* spp.) that are approx. 12 m high and 6 m apart. Macadamia fruits are not Medfly hosts. Norfolk Island pines (*Araucaria heterophylla* Franco; also nonhosts) in a few rows serve as wind breaks. The total area of IP under macadamia cultivation is approximately 390 ha. Just outside the mac nut orchard are fruit orchards, including papaya (*Carica papaya* L.) and guava (*Psidium* spp.), which are both Medfly hosts (APHIS et al. 2022). However, wild Medfly are not in the area based on previous surveys and trap capture from this study, likely because they have been outcompeted by *B. dorsalis* (Hendel) [Diptera; Tephritidae] and *Zeugodacus cucurbitae* (Coquillett) [Diptera; Tephritidae] (Wong et al. 1983, Vargas et al. 2016); (but see Vargas et al. 1995).

Weather conditions at IP were collected by NCEI (NOAA) reference weather station at Waiakea Research Station (station code “HI Hilo 5 S”) (Diamond et al. 2013). We used hourly datasets.

### Insects

Medflies used in these experiments were sourced from the colony at the USDA-ARS Daniel K. Inouye U.S. Pacific Basin Agricultural Research Center (DKI-PBARC) in Hilo, Hawaii. This colony was originally established around 1978 from wild Medfly collected on Oahu Island and has been maintained with periodic infusions of wild material. These flies are kept in large mixed-sex cages (0.6 × 1.18 × 1.32 m [*w* × *h* × *d*]) each holding about 50,000 individuals. There has been approximately one generation produced per week for the last 2 decades.

Medflies for each release were sterilized with X-rays using a Radsource 2400V (Radsource Technologies, Buford GA) irradiator in Hilo to administer a dose of 125 Gy following 2 to 3 h of hypoxia, 3 d before pupae eclosion. Irradiation was used to avoid introducing fertile pests to the area, which includes some orchards of host plants nearby. They were marked with fluorescent powder (green, orange, pink, or yellow; Day-Glo Color Co., Cleveland, OH) as pupae following Steiner (1965). Quality Control (QC) data, including pupal eclosion and flight ability, were collected prior to release for each cohort used (FAO et al. 2019). Adults were between 4 and 6 d postpupal eclosion at the time of release.

### Traps

**Fig. 4.**
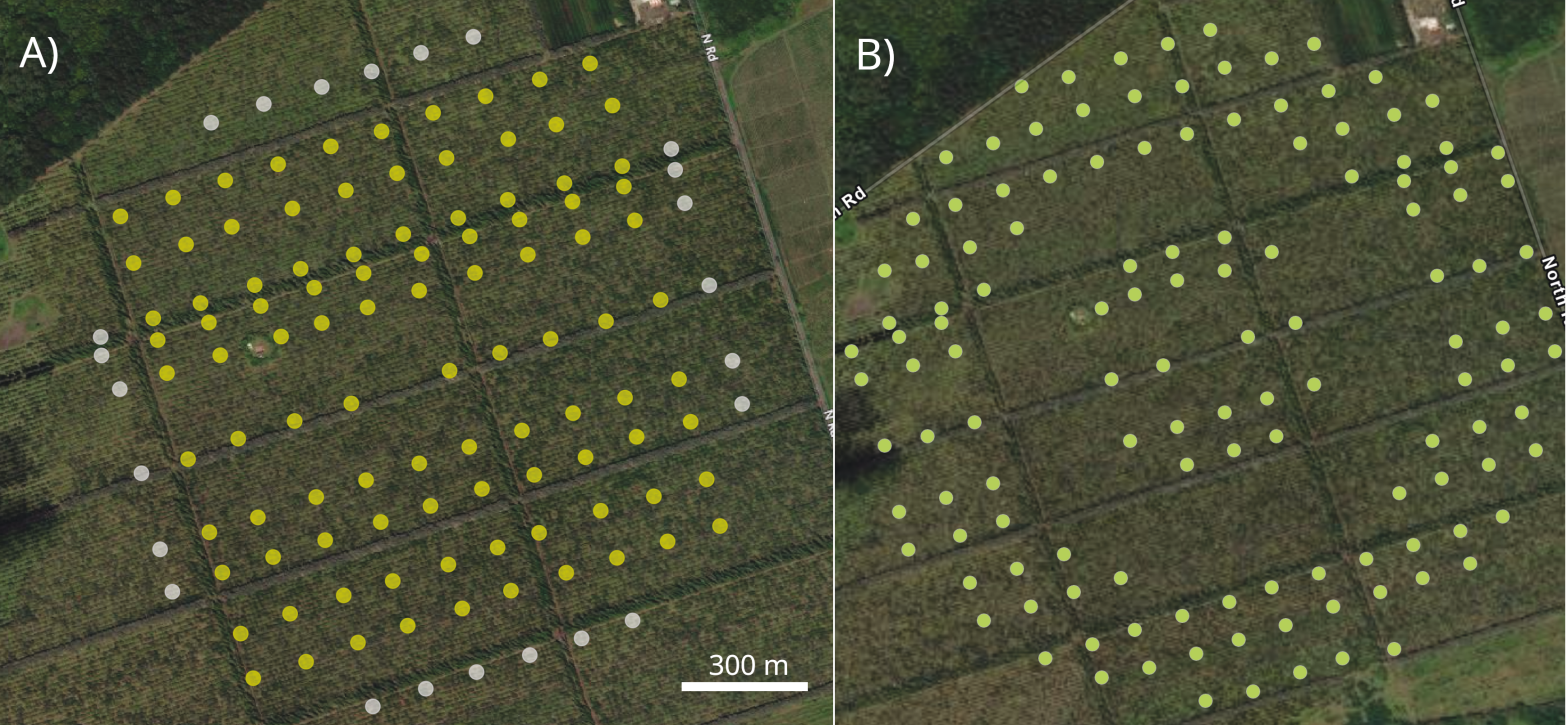
Trap locations in the macadamia nut orchard in Keaau, HI, for the A) FT grid and B) C&P designs. Standard trap locations and sentinel trap locations (FT grid only; outermost rows, darker points) are shown. Imagery: Mapping Hawaii, Maxar, Esri Community MapsContributors, Esri, HERE, Garmin, SafeGraph, GeoTechnologies,Inc, METI/NASA, USGS, EPA, US Census Bureau, USDA.

One day prior to the first release in a repetition, standard Jackson traps, the type used in detection networks in California (CDFA 2013), were baited with lure and set up on the study site in either the regular FT grid or the C&P design (Fig. 4). Traps were baited with 2 g trimedlure plugs (Scentry Biologicals, Billings MT). Plugs were hung from a basket-type holder within the Jackson trap, and a sticky insert was placed on the floor of the trap.

### Repetitions, Releases, and Services

Repetitions were set up every 4 wk alternating between the 2 treatment trap layout designs (C&P or FT), beginning 24 January 2022 and ending on 22 August 2022 (Table 2). The release and service intervals were selected to allow maximum collection of data from the deployed grid within the limits of available labor. The duration of the repetition was 2 wk followed by 2 wk of rest in-between treatments to allow insects from previous experiments to disperse or die before the next round. During each repetition we released flies on Tuesdays and Thursdays (4 total times) in rows between trees. Each release was marked with a different color of fluorescent dye powder. The traps were serviced on weekdays with no releases (6 times total; Table 2). On the last service day for each repetition most traps were removed from the field for the 2-wk rest period.

**Table 2.** Experiment dates in 2022 for setup, fly releases, and service days by repetition and treatment: C&P or FT

Repetition	1A	1B	2A	2B	3A	3B	4A	4B
**Treatment**	**C&P**	**FT**	**C&P**	**FT**	**C&P**	**FT**	**C&P**	**FT**
Set Up	1/24	2/21	3/21	4/18	5/16	6/13	7/11	8/8
Release 1	1/25	2/22	3/22	4/19	5/17	6/14	7/12	8/9
Service 1	1/26	2/23	3/23	4/20	5/18	6/15	7/13	8/10
Release 2	1/27	2/24	3/24	4/21	5/19	6/16	7/14	8/11
Service 2	1/28	2/25	3/25	4/22	5/20	6/17	7/15	8/12
Service 3	1/31	2/28	3/28	4/25	5/23	6/20	7/18	8/15
Release 3	2/1	3/1	3/29	4/26	5/24	6/21	7/19	8/16
Service 4	2/2	3/2	3/30	4/27	5/25	6/22	7/20	8/17
Release 4	2/3	3/3	3/31	4/28	5/26	6/23	7/21	8/18
Service 5	2/4	3/4	4/1	4/29	5/27	6/24	7/22	8/19
Service 6	2/7	3/7	4/4	5/2	5/31	6/27	7/25	8/22

Marked male flies were released between 8:30 and 10:00 AM at the center of the grid by opening a modified 24-gallon plastic container with a screened top and sides. Flies were allowed to disperse from the holding container, and then the remaining number of dead flies were recorded. Approximately 3,825 flying adult males were released each time.

Servicing the traps consisted of visually checking each Jackson trap, collecting any sticky inserts containing Medflies and replacing those with fresh inserts. Inserts with no flies were not removed. Inserts with Medflies were placed in brown paper bags and transported to the laboratory in Hilo. The inserts were processed by removing flies from the sticky card and placing them on filter paper. Their heads were then crushed with a small metal tool (approx. 5 mm diameter) dipped in acetone, which caused any dye in the ptilinum to dissolve and mark the filter paper. Flies were viewed under UV light with a dissecting microscope to determine the color of fluorescent dye powder marking the fly.

### Trapping Grids

In both treatments, intercolumn distances were consistently about 97 m, but interrow distances were more variable, with a minimum of about 50 m and a maximum of about 170 m (Fig. 4A and B). We manually calculated nearest trap distances (NTD) over all traps in both treatments and determined the mean, minimum, and maximum values.

#### Fully Trapped Grid Plus Sentinel Traps

The FT design had traps in 10 rows and 10 columns with a width (east–west) of ~975 m and a length (north–south) of ~950 m (Fig. 4A). Traps were placed at each row–column intersection, except for the release point, giving 99 traps total. Twenty of these within 275 m of the release point were “core area” traps. “Outer band” traps were 36 traps in the last rows on each side, while “noncore” traps were the 43 traps between those and the core. On each side an additional 6 “sentinel” traps were placed centrally (about 100 m) outside the outermost traps, to detect egressing insects, for 123 total traps.

#### C&P Grid

This grid consisted of 20 core area traps within −275 m of the release point (Fig. 4B). The perimeter held 108 traps, with an inner perimeter radius of about 575 m, and an outer radius of about 825 m. These distances were chosen to maximize the size of the C&P grid within the workable area of the orchard.

### Analysis

#### Spatial Point Patterns of Traps

We analyzed the spatial point patterns of each trapping design with the goal of visualizing and assessing and comparing how traps and captures were arrayed across the orchard in each treatment. In one analysis we plotted proportional traps by distance from the release point. We also determined kernel-smoothed densities (Baddeley et al. 2015) using the spatstat package in R (R Core Team 2022).

#### Captures

We summarized and evaluated Medfly captures in the 2 designs based on total, mean, and proportional captures by design and location (ie core and perimeter of C&P grid, core, noncore, outer band, and sentinels for FT grid). We also examined data for each release and service day. We compared treatment means of total flies captured using a least-squared means plot (“Fit Model”) in JMP Statistical Discovery (SAS Corporation, ver. 15.2.0; RRID:SCR_014242). We compared proportions of flies captured at common trap locations across treatments using the bivariate fitting procedure (“Fit Y by X”) with the paired *t*-test in JMP, and visually with a “points” plot from the spatstat R package. Our hypothesis was that captures in the FT design would be greater overall and more centrally located compared with the C&P design.

#### Captures Per Trap

We compared mean flies captured per trap by treatment using a least-squared means plot *n* JMP. We also compared treatments using regression of mean flies per trap from 0 to about 260 m (20 traps in both C&P and FT designs), which was the portion of the relationship that was linear (the metric was close to 0 and relatively constant for most distances beyond that). We used dummy variables to test shared or separate parameter values for the *y*-intercept and slope, using the “Fit model” process in JMP.

We also compared captures on a per trap basis for the FT outer band traps and the C&P perimeter traps to assess the likelihood of captures in each. We compared treatments in a least-squared means plot in JMP, with the hypothesis that captures in the perimeter of the C&P design should be lower than in the outer band. We calculated the total travel distance, *D*_Tot_, to each trap from the release point using the Haversine formula (Sinnott 1984), which accounts for the curvature of the earth. We evaluated the distributions of this metric by treatment and overall in JMP. Our hypothesis for the distance comparisons was that they would be similar between the grid designs, though larger values for the FT design might also be expected since the perimeter for that grid extended further.

#### Daily Dispersal Distance

For each captured fly we calculated the distance traveled per day, *D*_*Day*_, based on total distance and the number of days since release (ie service day minus release day). This estimate has some uncertainty, because some captures might have happened 1 or 2 d (maximum) before the service day. We evaluated the distributions of this metric by treatment and overall in JMP. Because this is an aspect of Medfly biology we did not expect this metric to vary between treatments.

#### Dispersal Densities

We fit histograms of proportional captures over both *D*_Tot_ and *D*_Day_. Bin sizes were determined with Sturge’s rule (Scott 2009). Because of trap placements, shorter distances were irregular, so the first bin was at either 150 or 160 m. We fit regressions to midpoints using the specialized, nonlinear modeling process in JMP with the 2-parameter exponential (decay) function, as follows:


$$\begin{array}{l} Y=a\times {e^{D}}^{i\times b} \end{array}$$ (1)

where *Y* is the proportion of flies (probability density), *a* is the scale (*Y*-intercept) parameter, *D*_i_ is the dispersal distance (total or daily), and *b* is the decay rate. Goodness of fit was evaluated using *R*^2^, and the *χ*^2^ probabilities for the estimated function parameters. We compared 95% confidence intervals for *a* and *b*.

We also compared the fit to a Cauchy distribution, which is sometimes preferred because it has a longer tail (eg Dominiak and Fanson 2023). The function was (Meats and Edgerton 2008):


$$\begin{array}{l} Y=a/\;\left[b\times \pi \times {\left(1\;+\;\left(D_{i}/b\right)\right)}^{2}\right]\; \end{array}$$ (2)

where *a* is a scale parameter and *b* is a rate parameter, as in Equation (1).

## Results

A data deposit containing all the primary data collected on recaptures per trap and date, as well as geocoordinates of each trap, and QC is available at “Ag Data Commons” (Manoukis et al. 2025).

### Trapping Grids

The mean NTD in the FG treatment was 84.3 m, with a minimum of 39.5 m, and a maximum of 160.4 m. In the C&P treatment, the mean NTD was 89.0 m, significantly greater than the FG mean (*P* < 0.05). For the C&P treatment, the minimum NTD was 36.5 m and the maximum was 117.1 m. The mean intertrap distance over all trap locations was 569.9 m for the FG treatment, but 807.9 m for the C&P, which were significantly different (*P* < 0.01). The increase for the C&P grid was due to the untrapped gap and the larger dimensions.

### Spatial Point Patterns

**Fig. 5.**
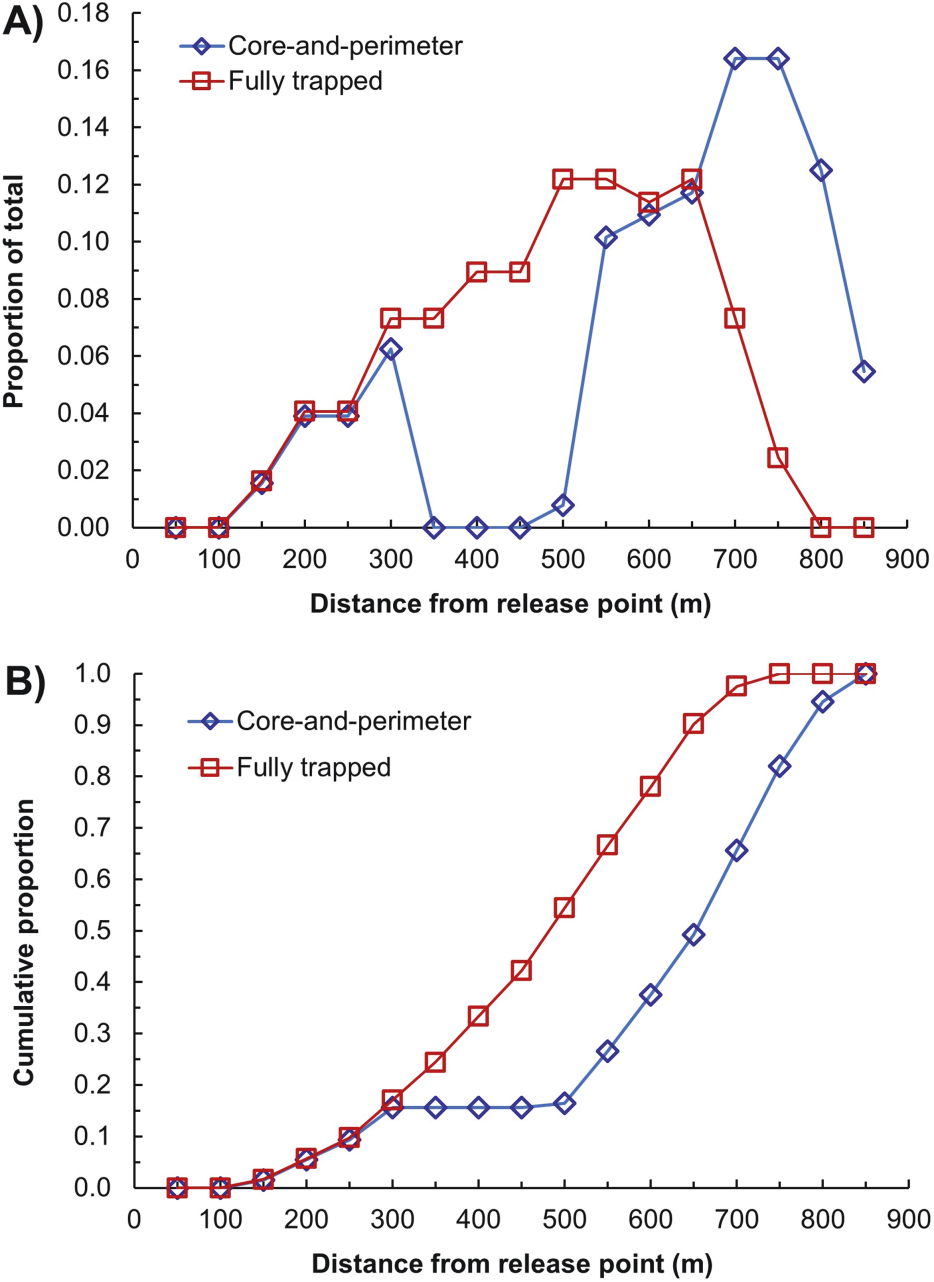
Trap proportions as a function of distance from the release point in 2 delimitation trapping designs, showing A) proportions of the total and B) cumulative proportions.

**Fig. 6.**
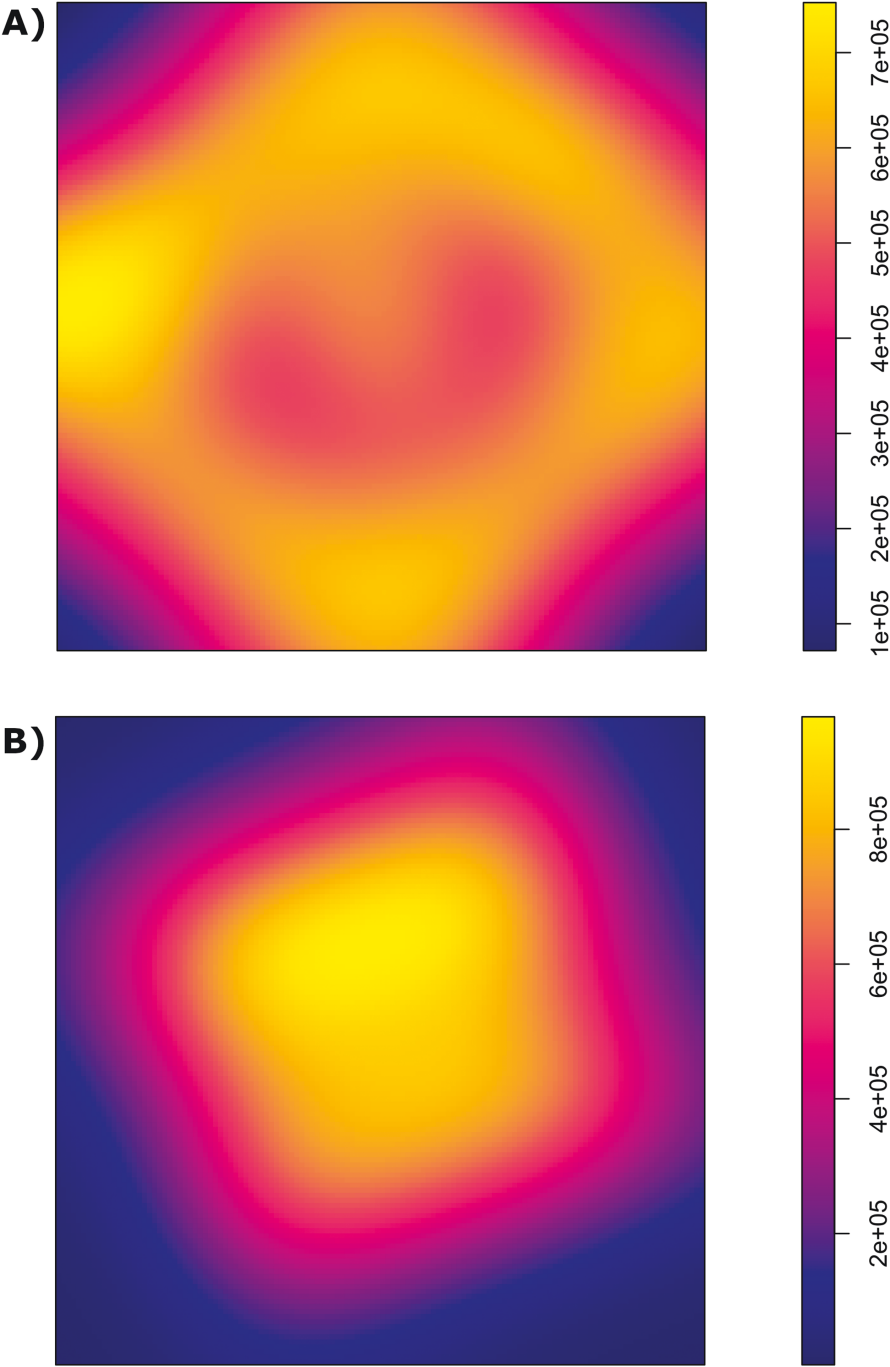
Kernel-smoothed density plots for standard trap locations in the A) C&P and B) FT grid delimitation designs in the experiment.

Trap densities within 300 m of the release points were similar between the 2 designs but deviated beyond that because of the untrapped area in the C&P (Fig. 5A and B). This gave the C&P design a greater kernel-smoothed density near the edges than in the center (Fig. 6A), whereas the density of the FT design was more uniform across the trapped area (Fig. 6B), as expected.

### Captures

#### Total Captures

Although total captures varied by trial (Table 3), we found no significant relationships with any weather-related variables (mean daily temperature, mean precipitation, etc.).

**Table 3.** Medfly captures in 2 trapping design treatments by replicate (Rep), showing total and proportional captures in different areas: core = innermost contiguous traps, perimeter (Perim.) = special outer ring of traps, outer = last row of standard traps in the FT grid, and sentinel = extra traps just outside the FT grid

Treatment	Rep	Flies captured (no.)					Proportions			
		**Core** ^a^	**Noncore** ^b^	**Outer** ^c^	**Sentinel**	**Total**	**Core**	**Noncore**	**Outer**	**Sentinel**
Core-and-Perimeter	1	1,364		27		1,391	0.981		0.019	
	2	781		16		797	0.980		0.020	
	3	436		25		461	0.946		0.054	
	4	906		72		978	0.926		0.074	
	Total	3,487		140		3,627	0.961		0.039	
Fully trapped grid	1	1,799	89	22	6	1,916	0.939	0.046	0.011	0.003
	2	557	97	43	5	702	0.793	0.138	0.061	0.007
	3	1,887	207	55	8	2,157	0.875	0.096	0.025	0.004
	4	772	109	25	5	911	0.847	0.120	0.027	0.005
	Total	5,015	502	145	24	5,686	0.882	0.088	0.026	0.004
**All**		8,502	502	285	24	9,313	0.913	0.054	0.031	0.003

^a^These were the 20 traps in each treatment that were set within 275 m of the release point.

^b^Traps in the FT design that were outside of the core but not in the outermost band.

^c^In the C&P design these traps were in the perimeter, while in the FT design these were the outermost band of traps.

More Medflies were captured in the FT than in the C&P treatment (Table 3). This was reasonable, because more than half of the traps in the FT design were within 500 m of the release point, compared to only 16% of traps for the C&P design (Fig. 5B). Despite that, the mean number of captures in the FT treatment of 1,421.5 was not significantly different from that in the C&P treatment of 906.8 (*P* = 0.256), most likely because of high variability (Table 3).

#### Captures Per Trap

Overall mean flies per trap for the FT treatment was 14.4, compared to 7.1 in the C&P treatment, but we found no significant difference (*P* = 0.115).

**Fig. 7.**
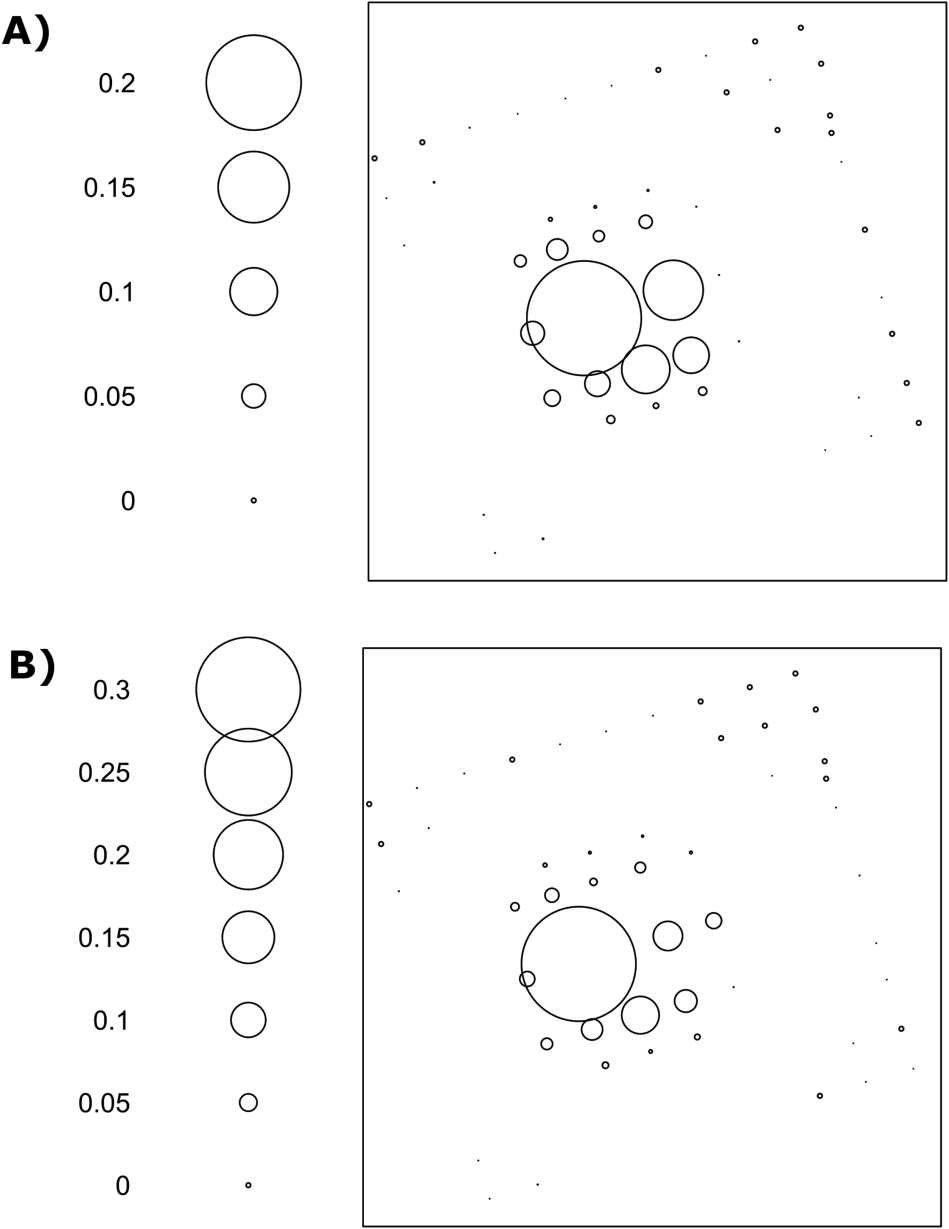
Point analysis of proportional Medfly captures in 51 common trap locations in: A) the FT grid or B) C&P designs.

**Fig. 8.**
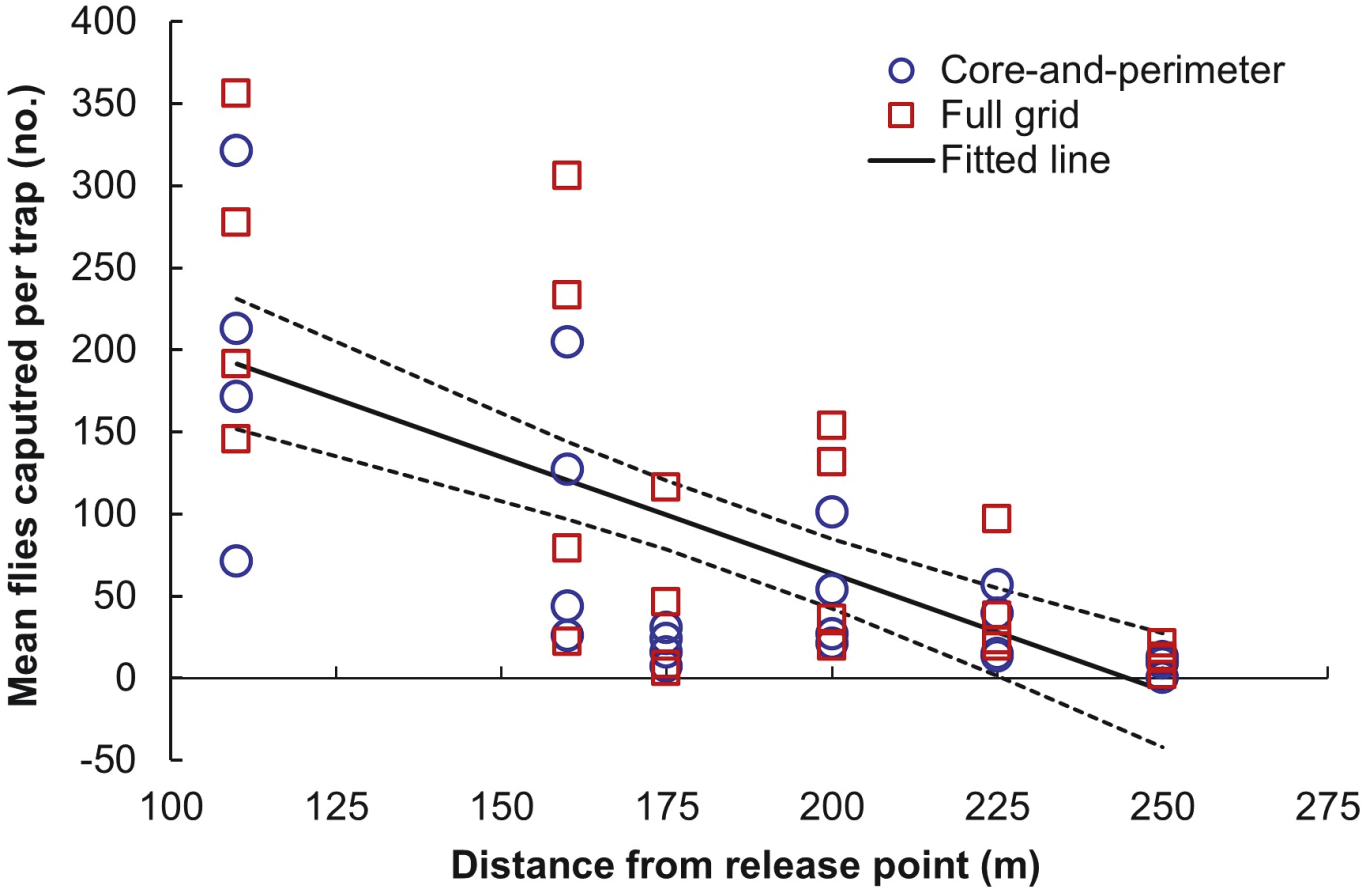
Mean number of Medflies captured per trap as a function of distance from the release point in field experiments, over a selected range (<260 m). Observed values (symbols) are shown for each treatment (grid design), as well as the final fitted regression line (black solid line) over both treatments, with 95% confidence intervals for mean predictions (dashed lines).

Numbers of captures at common trap locations did not differ spatially between the 2 treatments. Capture proportions at 51 common locations did not differ by paired *t*-test (*P* > 0.05), and a points analysis plot further demonstrated the similarity (Fig. 7). In addition, a linear regression was significant for mean flies captured per trap against distance from the release point up to 260 m (Fig. 8; *P* < 0.05; adjusted-*R*^2^ = 0.460), but regressions with treatment-specific parameters were not significant (*P* = 0.132).

Finally, mean flies per trap for the FT outer band traps was 1.07, and that was significantly greater than the value for C&P perimeter traps of 0.324 (*P* < 0.05). An outer band trap was 3.3 times more likely to capture a fly than a perimeter trap, despite there being many fewer of them.

#### Proportional Captures by Grid Area

On average, 88% or more captures occurred in the core areas of the 2 design treatments (Table 3). Perimeter captures in the C&P treatment were only about 2% to 7% of the flies captured in the 4 replicates, or 3.9% overall. A similar number of flies in total were captured in the outer band of the FT grid, but this was a smaller proportion (2.6%) because the total number of flies captured in that treatment was greater (Table 3). Very few flies were captured in the sentinel traps, but those captures verified that some Medflies could egress from the standard trapped area.

### Trap Usage

About two-thirds of the FT design traps captured flies on average, compared to a bit over a third of traps in the C&P design (Table 4). That was because only about 22% of perimeter traps in the C&P design captured flies. By comparison, the mean usage rate for the core area of the C&P was nearly 94%.

**Table 4.** Trap capture rates, expressed as numbers and proportions of traps with captures by treatment, replicate, and overall

Design	Area	Traps (no.)	Traps with detections (no.)						Proportion of traps with captures				
			Repetition					Mean	Repetition				Mean
			1	2	3		4		1	2	3	4	
FT	…	99	62	64		76	65	66.75	0.626	0.646	0.768	0.657	0.674
C&P	Core	20	20	18		19	18	18.75	1.000	0.900	0.950	0.900	0.938
	Perimeter	108	25	18		26	44	28.25	0.195	0.141	0.203	0.344	0.221
	All	128	45	36		45	62	47.00	0.352	0.281	0.352	0.484	0.367

### Daily Dispersal Distance

**Fig. 9.**
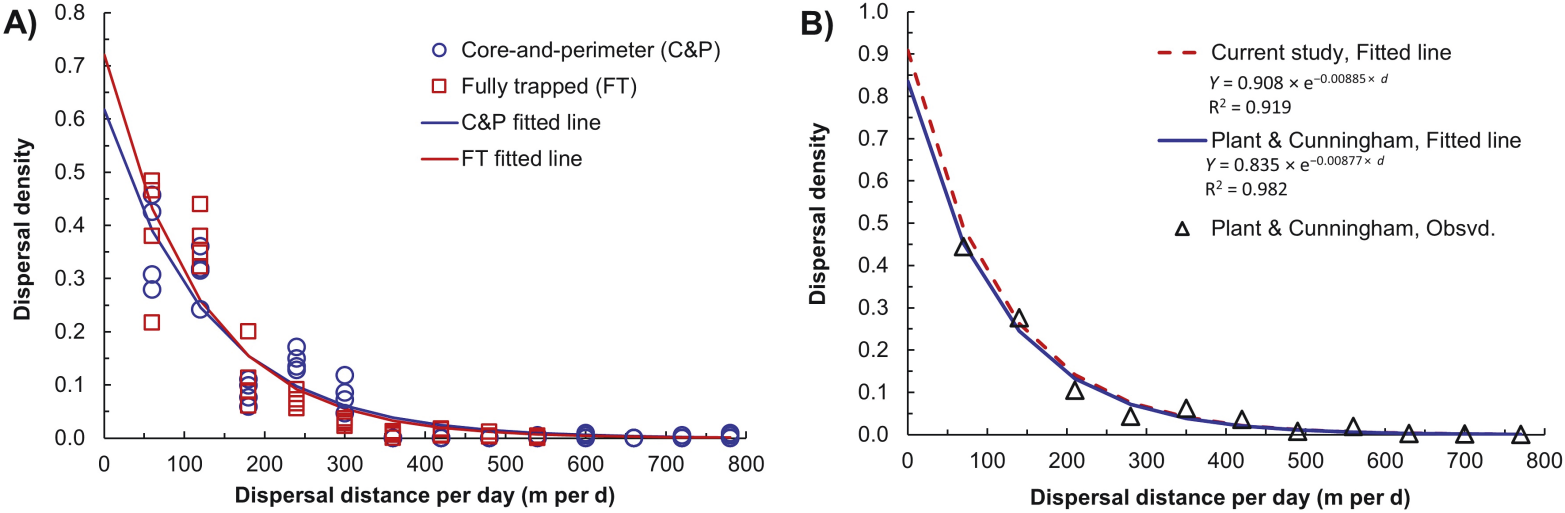
Dispersal density for Medfly as a function of daily dispersal distances for: A) observed data (empty symbols) for the current study by treatments and replicates, with fitted regressions (solid lines), and B) observed data (empty symbols) from Plant and Cunningham (1991), with a fitted exponential regression (solid line) and compared to a combined fit for the data in (A).

The range for *D*_Day_ was about 5 to 736 m, and the mean over all treatments was 96.3 m. Mean *D*_Day_ was significantly greater for the FT treatment compared with C&P (*P* < 0.05; not shown), but the difference was only 15 m. Fitted exponential decay curves for *D*_Day_ were very similar for the 2 treatments (Fig. 9A; statistically determined bin size = 60 m): confidence intervals for both parameters overlapped (Supplementary Table S1). The function fit from the combined data was used in subsequent simulations. The 50th percentile (median) of the distribution was 85.8 m, and the 99th percentile was 548.0 m. Also, the regression for the combined data was very similar to the fitted curve for the data from Plant and Cunningham (1991) (Fig. 9B).

### Total Dispersal Distance

**Fig. 10.**
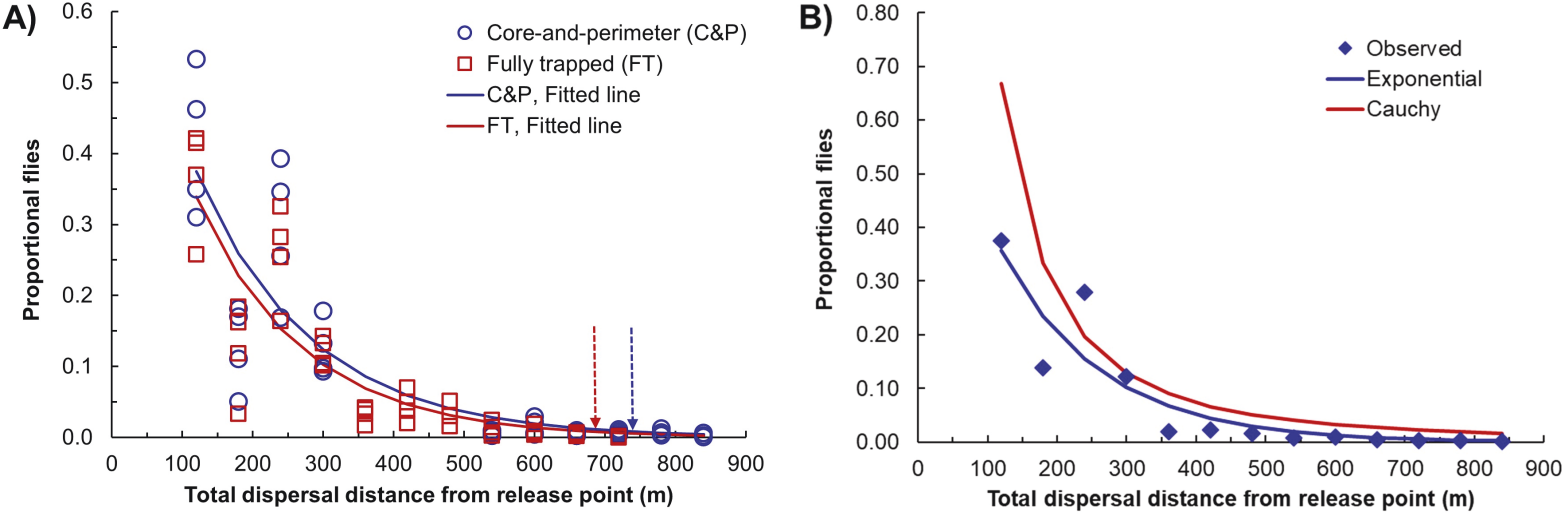
Probability distributions for total dispersal distance for Medfly in: A) 2 trapping design treatments and B) for the combined treatment data means, showing observed mean values, and fitted exponential decay and Cauchy curves. Both functions in (B) were fit to the replicate data in (A); mean values were shown for simplicity.

Maximum *D*_Tot_ in the FT treatment was 688.1 m (Table 5); the 2 traps with greater distance from the release point had zero captures. Maximum *D*_Tot_ in the C&P treatment was 833.1 m, which was the furthest trap from the release point. Both the median and mean *D*_Tot_ (*P* < 0.05) were greater for the FT treatment (Table 5), indicating that only a small proportion of flies were captured in the more distant traps in the C&P treatment. The fitted exponential decay curves of *D*_Tot_ for the 2 treatments were very similar (Fig. 10A; Supplementary Table S2). The regression for the C&P treatment had a slightly larger scale (*Y*-intercept) parameter, yielding percentile estimates that were about 8 percent longer than those for the FT treatment (Table 5).

**Table 5.** Total dispersal distances (*D*_Tot_) for Medflies in 2 trapping field experiment designs and overall, showing empirical medians, means, lower and upper 95% confidence limits, maxima, and percentiles from fitted regressions

Treatment	*D*_Tot_ (m)					*D*_Tot_ Percentiles (m)			
	Median	Mean	Lower	Upper	Maximum	5^th^	95th	99th	99.99th
Core-and-Perimeter	156.1	177.1	173.6	180.7	833.1	5.1	483.6	730.1	1,072.7
Fully trapped grid	180.2	190.8	188.3	193.3	688.1	7.0	448.6	678.7	989.1
Combined	169.2	186.5	184.4	188.6	833.1	7.3	425.1	642.3	949.6
*Difference* ^a^	24.1	13.7	14.7	12.6	145.0	1.9	35.0	51.4	83.6

^a^Absolute values between treatments.

The exponential decay function fit the data for *D*_Tot_ very well in all cases (Fig. 10A), and better than the Cauchy distribution based on MSE (Fig. 10B; Supplementary Table S3). The best regression was that for the combined data from FT and C&P designs.

Percentile distances from that function were shorter than the estimates from the treatment-specific functions, because of increased certainty in the estimate (Table 5). Ninetieth percentiles were relatively small, while 99.99th percentiles were about 1 km. Ninety percent of all distances were between 5.1 and 483.6 m in the C&P treatment and between 7.0 and 448.6 m for the FT treatment.

## Discussion

### Overview

The field trial results demonstrated operational differences between the 2 trapping grid designs. With large target pest populations, the FT design reliably produced lots of detections, mostly in the central area near the release point. Although smaller than the standard Medfly FT grid, flies still moved beyond the grid boundary, demonstrating one of the disadvantages of that approach. By contrast, about 95% of all captures in the C&P design were in the core, and perimeter captures were infrequent but significant. The core radius (575 m) was fully 2.7 km shorter than the recommended distance (Table 11; Caton et al. 2021a). Despite these differences in capture positions, estimated percentile distances for the 2 treatments were very similar (Table 5, Fig. 10A). In other words, the greater number of traps with the FT design were not required to accurately quantify Medfly dispersal. Although the trapping grid sizes were not large enough here to directly confirm that zero perimeter captures could occur in a C&P design, these results and the comparison of treatments for flies per trap in outermost traps (above) corroborated that relatively few flies would travel to the perimeter.

### FT Grid Design Disadvantages

We confirmed 2 previously mentioned disadvantages of the FT approach. The first was that the design usually used many more traps than necessary. In the FT treatment flies were captured in only 31% of all traps (not including sentinel traps) on average. In both treatments, over 96% of all detections occurred in the core areas on average (Table 3). Estimated 99.99th percentiles of about 1 km (Table 5) supported the idea that the standard FT grid design for Medfly with a side length of 14.5 km is greatly oversized, at least for delimitation (Caton et al. 2021a, 2021b).

The second disadvantage was that egress from a FT grid could go undetected. Although only 2.6% of all detections in the FT treatment occurred in the outer band of traps, flies were captured in sentinel traps in every trial (Table 3). Hence, captures in the outer band of a FT design are not reassuring but alarming. Although an insect can also escape past the perimeter of a C&P design, greater (optimized) distance from the epicenter makes this less likely than with a traditional FT grid. In this trial, with a shorter inner radius than recommended, flies were more than 3 times less likely to be captured in the C&P perimeter than in the FT outer band. Lack of relevant biological information and safety against undetected egress by insects is likely why many surveys and quarantine areas seem to have been oversized (Meats 2014, Dominiak and Fanson 2020, Caton et al. 2021a).

The square shape of nearly all published FT delimitation plans is an additional flaw that reduces efficiency (Caton et al. 2021b, Fang et al. 2022). Circular designs are better.

### Dispersal Findings

The level of agreement between the fitted curves for *D*_Day_ between this experiment and the Plant and Cunningham (1991) study was remarkable (Fig. 9B). The similar origin of the flies and the testing environment likely contributed to this, but it also suggested consistent behavior in the species. Greater mean *D*_Day_ for the C&P treatment than the FT treatment was reasonable because in that design captures could be more distant.

Results confirmed that Medfly is only moderately motile (Weldon et al. 2014, Caton et al. 2021a). It may seem that mean *D*_Day_ of about 100 m should result in significant travel distances over time, but mean *D*_Tot_ in the experiment here was only about 200 m, and 99th percentiles from regressions were about 700 m (Table 5).

Dispersal of Medflies in this study may have been limited relative to wild flies for at least 2 reasons: We used colony-reared insects in this study, and they were sterilized before release. Wild collected or “wildish” (less than F4) tephritids generally display more varied behavioral and survival phenotypes than colony-reared insects (Manoukis and Gayle 2016). For example, response of wild male Medfly to the lure trimedlure relative to that of colony-reared males has been found to be similar (Barry et al. 2003), lower (Wong et al. 1982) or higher (Shelly and Edu 2009). Comparison between studies is complicated by variances in experimental design and between colony strains used. For Medfly, specifically, the colonization process is known to affect many factors, including developmental rates (de Souza et al. 1988, Vargas et al. 2000), which might have an indirect effect on dispersal via timing and duration of movement. The flight ability tests (using “flight tubes”) used in the mass rearing (Boller et al. 1981) may not show differences between wild material and colony insects (Economopoulos 1992), but these cannot easily be related to movement across a landscape (Steyn et al. 2016).

The effect of irradiation on dispersal has been most directly examined by Wong et al. (1982) and in subsequent studies by Gavriel et al. (2012) and Al-Gerrawy et al. (2021). In aggregate these studies show that irradiation reduces flight distances, though likely not the pattern of distribution of the insects in the area covered. In addition, survival is decreased by irradiation (eg Guerfali et al. 2011), which will limit life time dispersal distance. So, the results here might be considered a lower bound of likely Medfly movement in the case of a wild, unirradiated individual.

Still, our findings were similar to numerous results from earlier published Medfly trapping analyses, such as distance limits of around 1 km in Meats et al. (2003) and 700 m in Meats and Smallridge (2007). Weldon et al. (2014) list several additional Medfly dispersal studies, with most reporting total dispersal distances of less than 1 km. Meats (2014) even suggested that detections of *any* fruit flies beyond 1.6 km should be treated as separate introduction sites. Factors affecting the dispersal capability of Medfly could include moderate daily dispersal probabilities (Caton et al. 2024), high mortality (eg Plant and Cunningham 1991), and variable directionality (Miller et al. 2022).

In conclusion, while the experimental site was too small to directly test the worthiness of the C&P design, the results were supportive. Medflies were unlikely to travel to a distant perimeter and much more likely to be trapped around the population epicenter. The difference in those likelihoods would only increase with smaller, adventive population sizes, and the larger grid areas needed for a real incursion. FT grids seem certain to produce more detections than a C&P design, but that data did not improve or even change estimates of dispersal distances or occupancy (Table 5). At these sizes the C&P treatment used *more* traps than the FT treatment but will use less if the inner radius is 3.2 km or longer (Table 1). Medfly dispersal was remarkably consistent across treatments, which was expected, and *D*_Day_ was strongly corroborated by a previous study. The exponential function fitted dispersal distances well, and better than the Cauchy distribution for *D*_Tot_ (Fig. 10B). Results confirmed that Medfly is moderate in the continuum of fruit fly dispersal abilities, and provided details that will facilitate model evaluations of the C&P design. In combination with the simulation results in Caton et al. (2025) we believe program managers have the information needed to assess the viability and desirability of C&P designs for delimitation programs against new invasive pest insects.

## Supplementary material

Supplementary material is available at *Journal of Economic Entomology* online.

toaf095_suppl_Supplementary_Tables_S1-S3

toaf095_suppl_Supplementary_Videos
