# Supplementary material for: Evaluating a novel core-and-perimeter delimiting trapping survey design for insects. I. Field experiment
Source: J Econ Entomol. 2025 Jun 14;118(4):1621–33. doi: 10.1093/jee/toaf095 (PMC12397972; doi:10.1093/jee/toaf095)
Supplement: toaf095_suppl_Supplementary_Tables_S1-S3 [file toaf095_suppl_supplementary_tables_s1-s3.docx]

# Supplementary material for **Evaluating a Novel Core-and-Perimeter Delimiting Trapping Survey Design for Insects: I. Field Experiment**

Barney P. Caton, Hui Fang, Ernie Hain, Nadya Kandel, Rosalie Nelson, Godshen R. Pallipparambil, and Nicholas C. Manoukis

**Table S1**. Regression results for Medfly dispersal distance per day by treatment and overall, showing regression mean squared errors, parameter estimates and standard errors for the estimates.

| **Treatment** | **Regression MSE** | **Parameter** | **Estimate** | **SE** |
| --- | --- | --- | --- | --- |
| C&P | 0.00186 | *a* | 0.618 | 0.0499 |
|  |  | *b* | -0.00770 | 0.000687 |
| FT | 0.00407 | *a* | 0.721 | 0.0835 |
|  |  | *b* | -0.00857 | 0.00107 |
| Overall | 0.00239 | *a* | 0.908 | 0.0590 |
|  |  | *b* | -0.00885 | 0.000591 |

**Table S2**. Regression results for Medfly total dispersal distance by treatment and overall, showing regression mean squared errors, parameter estimates and standard errors for the estimates.

| **Treatment** | **Regression MSE** | **Parameter** | **Estimate** | **SE** |
| --- | --- | --- | --- | --- |
| C&P | 0.00536 | *a* | 0.783 | 0.157 |
|  |  | *b* | -0.00615 | 0.00113 |
| FT | 0.00342 | *a* | 0.750 | 0.121 |
|  |  | *b* | -0.00662 | 0.000875 |
| Overall | 0.00433 | *a* | 0.779 | 0.0991 |
|  |  | *b* | -0.00648 | 0.000708 |

**Table S3**. Regression results for Medfly mean total dispersal distances, for the exponential decay and the Cauchy function, showing regression mean squared errors, parameter estimates and standard errors for the estimates.

| **Treatment** | **Regression MSE** | **Parameter** | **Estimate** | **SE** |
| --- | --- | --- | --- | --- |
| Exponential | 0.00259 | *a* | 0.828 | 0.230 |
|  |  | *b* | -0.00699 | 0.00154 |
| Cauchy | 0.00298 | *a* | 634.554 | 252.075 |
|  |  | *b* | 59.175 | 52.276 |
